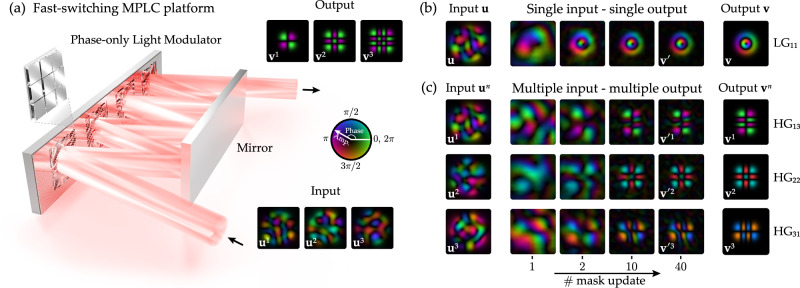# Author Correction: Self-configuring high-speed multi-plane light conversion

**DOI:** 10.1038/s41467-026-69974-0

**Published:** 2026-03-23

**Authors:** José C. A. Rocha, Unė G. Būtaitė, Joel Carpenter, David B. Phillips

**Affiliations:** 1 Physics and Astronomy, University of Exeter, Exeter, EX4 4QL UK; 2 School of Electrical Engineering and Computer Science, The University of Queensland, Brisbane, QLD 4072 Australia

**Keywords:** Adaptive optics, Fibre optics and optical communications, Imaging and sensing

Correction to: *Nature Communications* 10.1038/s41467-025-66798-2, published online 08 December 2025

In Fig. 1c of this article the colour scheme of the bottom right panel was incorrect; the original and corrected figure panels are shown below. The original article has been updated.

The incorrect version of Fig. 1 is:
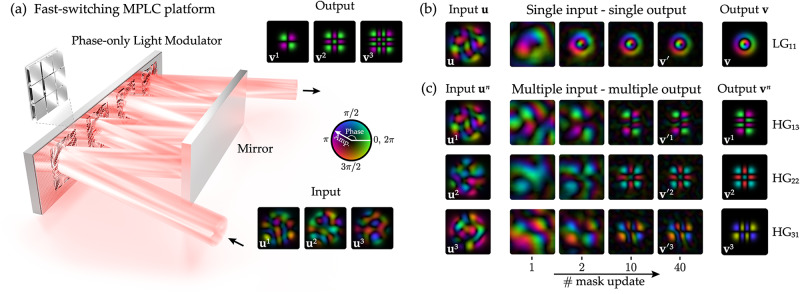


The correct version of Fig. 1 is: