# Supplementary material for: Self-configuring high-speed multi-plane light conversion
Source: Nat Commun. 2025 Dec 8;17:73. doi: 10.1038/s41467-025-66798-2 (PMC12770535; doi:10.1038/s41467-025-66798-2)
Supplement: Supplementary file 1 — Supplementary Information [file 41467_2025_66798_MOESM1_ESM.pdf]

Self-configuring high-speed multi-plane light conversion

Supplementary information

§1: Fidelity progression

Supplementary Fig. 1 presents how fidelity of the output evolves with each mask update, for the light shaping experiments shown in main paper Fig. 1. We define fidelity f as:

$$f = \left| \sum_i \mathbf{v}_i \mathbf{v}_i'^* \right| , \tag{1}$$

where i indexes pixels, * indicates a complex conjugate, and $\mathbf{v}$ and $\mathbf{v}'$ have been normalised to have the same power. Here $\mathbf{v}'$ has been experimentally obtained using digital holography.

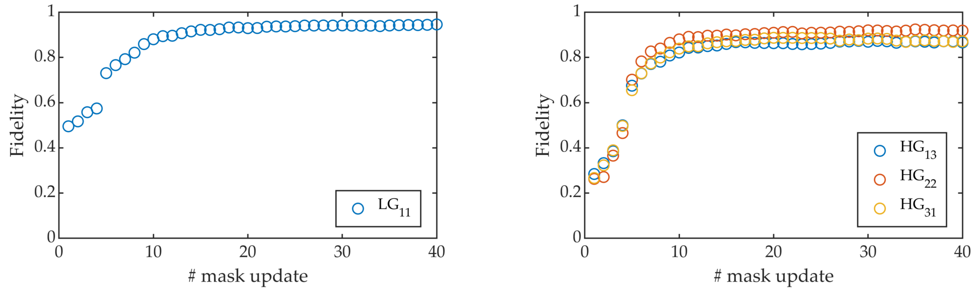

Supplementary Figure 1. **Fidelity progression during self-configuring MPLC optimisation.** Data on the left corresponds to main paper Fig. 1(b), and data on the right – to main paper Fig. 1(c).

§2: Comparison of in-situ optimisation to wavefront matching

Here we compare the performance of our self-configuring MPLC algorithm and the wavefront matching (WFM) method. Supplementary Fig. 2 presents simulation results for sorting 10 HG modes using 4 phase masks, each $n_{\text{pix}} = 256$ pixels in width. We can see that the self-configuring algorithm approaches the performance of the WFM method as the number of modes (here plane-waves) is increased. If the WFM method is limited to the same 10000 plane-wave components, the two algorithms yield the same results - after 24 mask updates the self-configuring algorithm reaches mean total crosstalk of 6.40%, while the WFM method reaches 6.38% (the unrestricted WFM method achieves 4% in the same number of iterations). Likewise, in terms of design efficiency, η_{design}, the self-configuring and restricted WFM methods reach mean mode transformation efficiencies of $\eta_{\text{design}} = 37.0\%$ and $\eta_{\text{design}} = 36.8\%$ respectively (the unrestricted WFM method achieves $\eta_{\text{design}} = 40\%$).

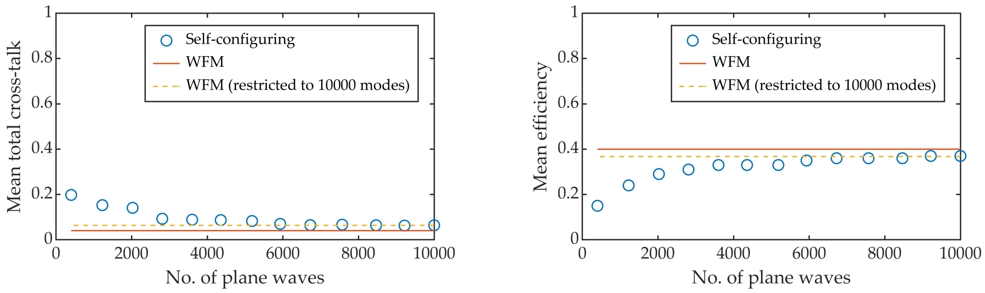

Supplementary Figure 2. **Comparison of self-configuring and wavefront-matching methods.** Here we simulate sorting of 10 HG modes, with 4 planes (each 256 pixels across).

§3: Demonstration of in-situ MPLC optimisation at 1.44 kHz and polarisation invariance

Here we present results for a 5-mode 4-plane speckle sorter optimised with the PLM running at 1.44 kHz. The MPLC was optimised for vertical polarisation, and then tested for both vertical and horizontal polarisations with negligible differences in performance, as can be seen in the cross-talk matrices in Supplementary Fig. 3. The average cross-talk for vertical polarisation

is -18.0 dB, and for horizontal polarisation it is -17.8 dB.

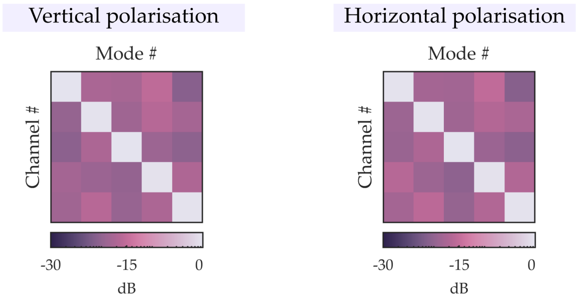

Supplementary Figure 3. **Cross-talk matrices for a 5-mode speckle sorter at two orthogonal polarisations.**

§4: Estimation of MPLC efficiency
Here we consider the efficiency of the transformation enacted by the MPLC. The overall efficiency, η, is given by

$$\eta = \eta_{\text{design}}\eta_{\text{exp}} = \eta_{\text{design}}(r_{\text{SLM}}d_{\text{SLM}})^M, \tag{2}$$

where η_{design} is the theoretical design efficiency (assuming each phase mask is ideal and so lossless), and η_{exp} is the experimental efficiency of the physically realised MPLC, which is separated into the product of two contributions:
- Reflection efficiency, r_{SLM}, defined as the percentage of incident light reflected from the PLM that is sent to the zero diffraction order when the PLM displays a flat pattern;
- Diffraction efficiency, d_{SLM}, defined as the percentage of the zero order light that can be controllably diffracted to the first order, when the PLM displays a phase ramp. The diffraction efficiency can be modelled [1], and decreases for steeper phase ramps, so here we choose a value of $d_{\text{SLM}} = 0.84$ based on the typical spatial frequencies of the displayed phase patterns in our MPLC designs.

Estimate of efficiency of current prototype: We directly measure the overall efficiency η, which, as shown in Supplementary Eq. 2, depends upon both the design efficiency of the target transformation η_{design}, and the experimental efficiency η_{exp}. The efficiency is measured by capturing the total intensity of the MPLC input and output beams by summing the pixels of images recorded with a camera, and calculating the ratio of these intensities. Supplementary Table I shows the results of these measurements for the simplest case: an HG sorter that just transforms a single input Gaussian mode (HG_{00}) to a focussed spot at the output (top row), and for a 3 HG mode sorter (bottom row). In each case we also calculate the expected design efficiency using a simulation of our experiment. This enables an estimate of the experimental efficiency to be calculated, by rearranging Supplementary Eq. 2: $\eta_{\text{exp}} = \eta/\eta_{\text{design}}$. We analyse two cases: firstly, in which the overall efficiency is calculated by considering only the total intensity of light transmitted into a disk specifying the output channel (referred to as 'in channel'). As this measurement is sensitive to the radius of the specified channel, we also compare this to the efficiency calculated by considering all output light that reaches the camera (referred to as 'all image').

We measure overall efficiencies ranging from $\eta \sim 2 - 7\,\%$. Losses are dominated by the experimental efficiency, which we estimate to be in the range $\eta_{\text{exp}} \sim 3 - 8\,\%$. These values agree with recent studies of PLM reflectivity [2] and diffraction efficiency [3] at visible wavelengths, which have found $r_{\text{SLM}} \sim 0.63$ and $d_{\text{SLM}} \sim 0.84$, yielding estimated experimental efficiencies of $\eta_{\text{exp}} \sim (0.63 \times 0.84)^4 \sim 0.08$ in our $M = 4$ plane MPLC.

Projections of future efficiency improvements: We now consider possible future increases in efficiency based on projections of how much the reflection efficiency might be improved in future systems. We assume that diffraction efficiency will remain fixed at $d_{\text{SLM}} \sim 0.84$. We give two examples:
(1) A near infra-red-optimised PLM is currently under development by TI (recently introduced in a presentation at SPIE Photonics West conference in January 2025 [1], but not yet available for testing), which is designed to operate across the wavelength range $730 < \lambda < 1630\,$nm, and is expected to have an improved reflection efficiency of $r_{\text{SLM}} \sim 0.8$. Therefore, for an $M = 4$ plane MPLC, assuming a reflection efficiency of $r_{\text{SLM}} = 0.8$ suggests an experimental efficiency of $\eta_{\text{exp}} = (0.8 \times 0.84)^4 \sim 0.2$.
(2) Liquid crystal SLMs have undergone several decades of development, and their reflection efficiency has been enhanced using narrowband anti-reflection coatings of the cover-glass, and also dielectric backplane mirrors. These features grant liquid crystal

SLMs a reflection efficiency of up to $r_{\mathrm{SLM}} \sim 0.95$ (e.g., the Holoeye PLUTO-2.1-NIR-113 has a quoted reflectivity of 95% – see specification here: https://holoeye.com/products/spatial-light-modulators/). These high efficiencies have already enabled the implementation of liquid crystal SLM-based MPLCs of up to ten planes [4]. Therefore, we estimate that for an $M = 4$ plane MPLC, assuming a reflection efficiency of $r_{\mathrm{SLM}} = 0.95$ suggests an experimental efficiency of $\eta_{\mathrm{exp}} = (0.95 \times 0.84)^4 \sim 0.4$.

No. of HG modes	η_{design} (sim.)	η (meas., in channel)	η (meas., all image)	η_{exp} (est., in channel)	η_{exp} (est., all image)
1	0.93	0.04	0.07	0.04	0.08
3	0.64	0.02	0.05	0.03	0.08

Supplementary Table I. **MPLC efficiency**.

§5: Dependence of the algorithm on the initial mask phase

Here we consider how the performance of the self-configuring MPLC algorithm depends on the initial state of the phase masks. We run the optimiser (in simulation) 40 times, each starting with a set of four masks with a random phase distribution.

In each instance the algorithm converges to a slightly different set of phase masks, but the performance is largely the same, as can be seen in Supplementary Fig. 4. In comparison, if the initial masks have a flat phase (orange cross in Supplementary Fig. 4) the results are quite similar for cross-talk, but the efficiency increases from ~ 0.2 to ~ 0.25.

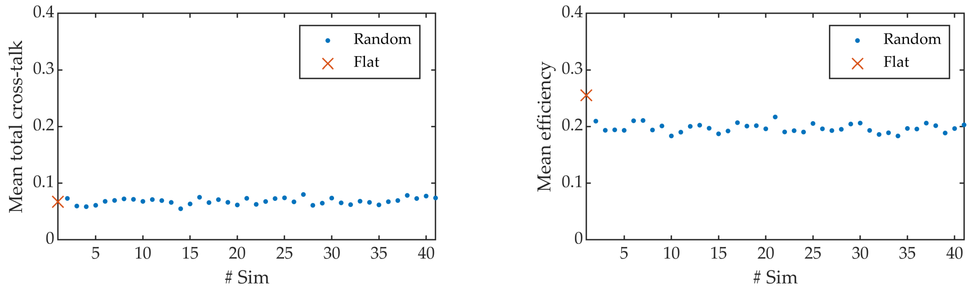

Supplementary Figure 4. **Simulated sorter performance for different initial mask phase.** Here we simulate sorting of 10 HG modes, using 4 planes (each 256 pixels across), a set of 4096 plane-waves as basis for each TM, for 5 cycles (i.e., 20 mask updates).

§6: How sorter performance scales with the number of masks

In simulation, we evaluated the performance of the self-configuring algorithm for different number of planes in the design. In Supplementary Fig. 5 we show the performance of MPLC designs which sorts 10 HG modes and 7 orthogonal speckle fields, with a variable number of planes (each 256 pixels across), a set of 4096 plane-waves per TM, for 40 mask updates in total. For the HG sorter, we see that although there is improvement in MPLC cross-talk and efficiency beyond 4 planes, it is relatively gradual, thus requiring many more planes to significantly improve the MPLC performance. More generally, the number of planes M needed to efficiently enact an arbitrary transform on N modes typically scales linearly with N [5].

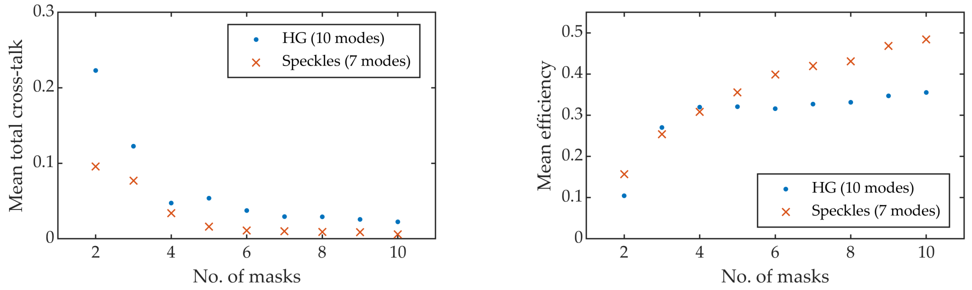

Supplementary Figure 5. **Simulated sorter performance for different designs and number of masks.**

§7: Choice of basis in which TM is measured

In addition to the plane-wave basis discussed in the main paper we have investigated two alternatives - Hadamard basis and discrete cosine transform (DCT) basis [6]. We note that since the DCT basis modes have a spatially varying amplitude as well

as phase, we cannot use them directly and have therefore employed the following strategy: When measuring the TM we take the phase of each DCT basis function and display that on the masks as an approximation of the ideal basis modes. When constructing the new phase mask, we use the complete DCT mode description.

Supplementary Fig. 6 compares the masks that the algorithm converged to and their performance when sorting 10 HG modes. The plane-wave basis achieves the best results, especially in terms of efficiency, while the Hadamard and DCT bases show similar performance. The results are summarised in Supplementary Table II. We conjecture that the plane-wave basis leads to the best results because, when measuring a TM with fewer measurements than there are PLM pixels on each plane, the plane-wave basis converges to smoothly varying phase masks – which have also been demonstrated to be optimal for wavefront shaping within forward scattering scenarios such as ours [7, 8]. In contrast, the Hadamard basis will converge to coarsely pixellated phase mask designs which have sharp edges that will scatter light at high angles out of the optical system.

The DCT basis also produces smooth masks which, however, do not have equally good performance. This is because we cannot accurately represent the basis functions in our phase-only implementation, as discussed above, which introduces errors that reduce the fidelity of the final design. We also note that most of the light intensity reflecting from each plane is situated in the middle of the masks, which explains the "noisy"-looking phase distribution close to the edges of the masks, where there is minimal light and so these areas make negligible contribution to the transformation and have phase values which are not well-defined.

Basis	Cross-talk	Efficiency
Fourier (plane-waves)	0.07	0.26
Hadamard	0.09	0.13
Discrete cosine transform	0.10	0.14

Supplementary Table II. **MPLC efficiency for different Transmission-Matrix bases.**

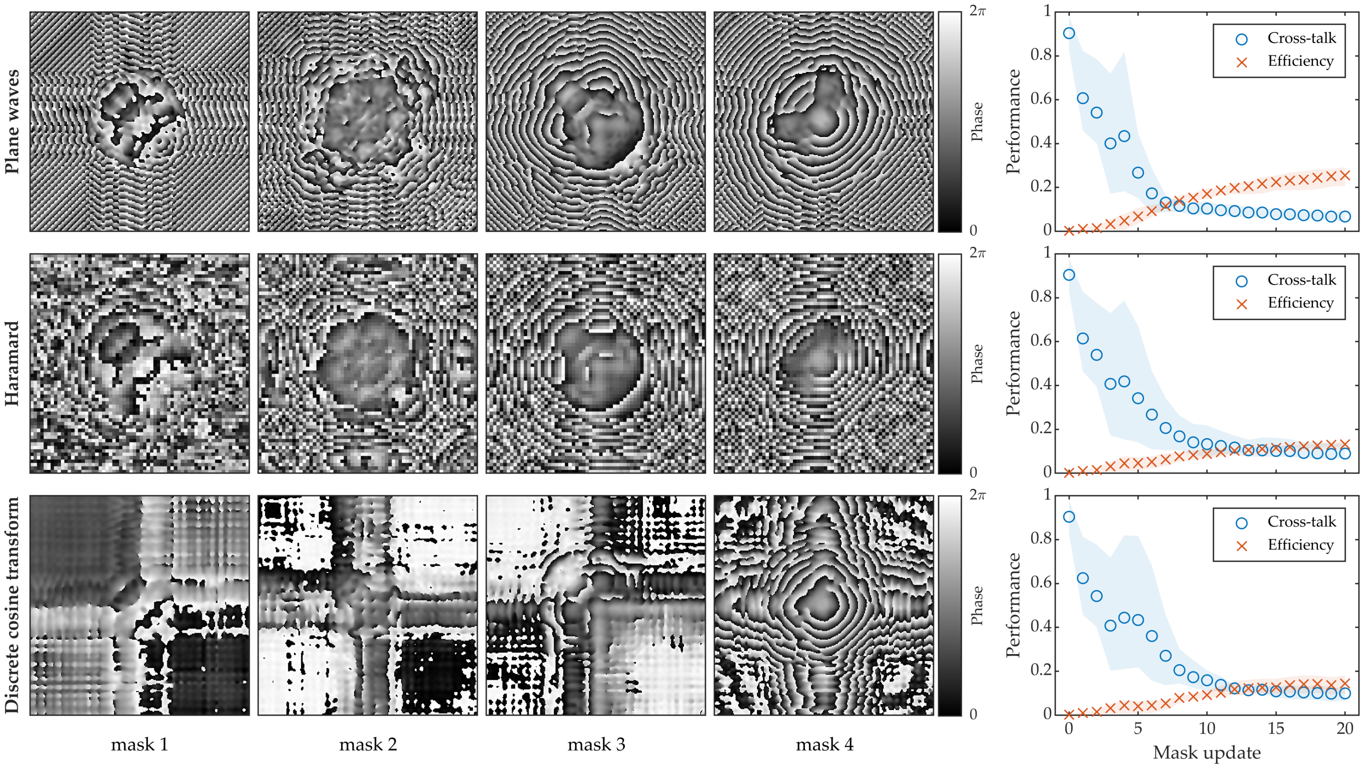

Supplementary Figure 6. **Sorter masks and performance when different bases are used for measuring the TM.** Here we simulate sorting of 10 HG modes, with 4 planes (each 256 pixels across), a set of 4096 modes for measuring the TM, for 5 cycles (20 mask updates).

§8: Sorting performance in the presence of misalignments

MPLC performance is notoriously sensitive to unknown misalignments or phase curvature of the planes. To illustrate this sensitivity, here we explore (in simulations) how quickly the performance of a sorter degrades in the presence of such imperfections.

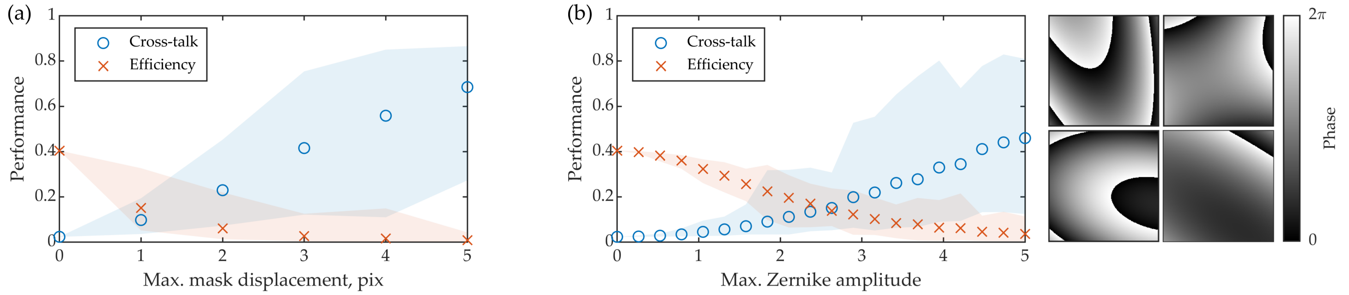

Supplementary Figure 7. **Effect of misalignment on sorting performance.** How cross-talk and efficiency of a simulated mode sorter change when (a) the masks are displaced and (b) a phase distortion is added to the masks – an example of phase distortion, with a maximum Zernike amplitude of 3, is shown on the right. Each data point was obtained from 100 repeated simulations and the shaded regions indicate the full range of results from these simulations. Here we simulate sorting of 10 HG modes, using 4 planes (each 256 pixels across).

First, we consider mask misalignment in the transverse plane. We take the masks of a 10-mode HG sorter which were designed using the WFM method, and displace them along x and y dimensions by a randomly chosen number of pixels, with some fixed maximum displacement. Since randomness is involved, we repeat each simulation 100 times to get a representative average. The change in cross-talk and efficiency with increasing maximum mask displacement are shown in Supplementary Fig. 7(a), where it can be seen that displacements by even a single pixel (on average) have a substantial effect on MPLC performance.

We also consider what happens if the modulator with which the masks are created (be it a liquid-crystal SLM or a PLM) is not perfectly flat. To model this, we generate a random weak phase aberration across the mask surface by adding five low-order Zernike modes (tip, tilt, defocus, oblique astigmatism, and vertical astigmatism) with random magnitudes, with some fixed maximum magnitude. This aberration is then added to the original masks and their performance is evaluated. Again, we obtain representative averages from 100 simulations for each maximum Zernike magnitude. The effect on cross-talk and efficiency along with an example of simulated aberrations can be seen in Supplementary Fig. 7(b).

Although challenging to do so, it is, in principle, possible to manually align an MPLC to correctly match the alignment degrees of freedom with the physical model used to design the MPLC. Therefore, considering only misalignments, we would expect the performance of our in-situ optimiser to be equivalent to a perfectly manually aligned MPLC (as long as the signal-to-noise ratio of the in-situ measurements is high, and enough TM samples were used – see SI §2). However, if the planes feature unknown aberrations – that are therefore not included in the physical model used to design the MPLC offline – a manually aligned MPLC will always suffer reduced performance in comparison with our in-situ optimisation scheme, which can automatically account for these unknown aberrations, and so will surpass the performance achievable through manual alignment.

We further investigate (in simulation) if the above discussed loss in performance caused by misalignment can be corrected by using our self-configuring algorithm. In this scenario, we start the self-configuring algorithm with pre-calculated phase masks which have been displaced from their ideal position by up to four pixels. In Supplementary Fig. 8 we see that both cross-talk and efficiency converge in fewer mask updates if optimisation is started with the pre-calculated but misaligned masks, than if the optimisation is started with flat phase masks. This suggests that first making best efforts to align a pre-designed MPLC, followed by running our self-configuring approach, is a promising way to enhance MPLC performance.

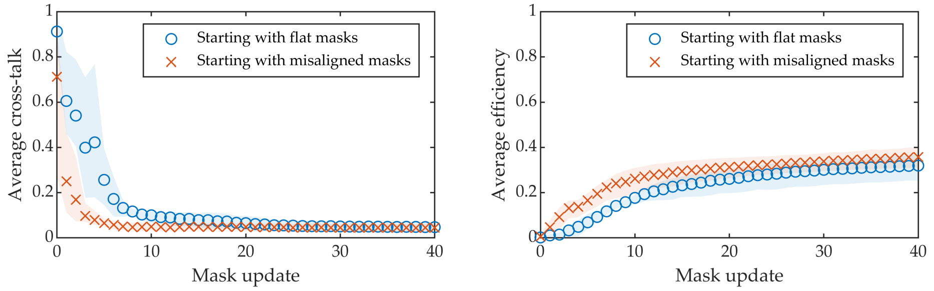

Supplementary Figure 8. **Correcting a misaligned pre-calculated sorter with the self-configuring algorithm.** Average cross-talk is shown on the left and average efficiency on the right. Shaded regions indicate the spread of values for individual modes. Here we simulate sorting of 10 HG modes, using 4 planes (each 256 pixels across), with a set of 4096 plane-waves as basis for each TM.

§9: Self-configuring optimisation performance with 105 modes

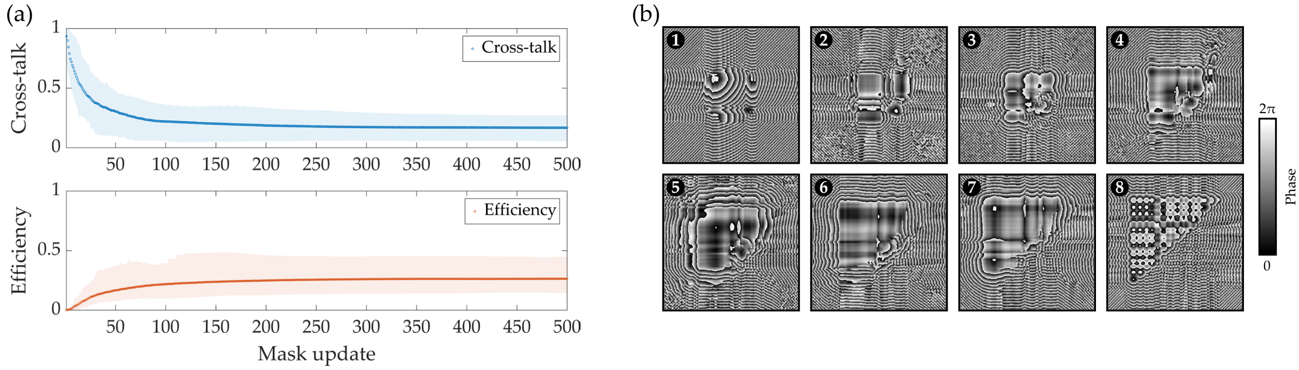

Supplementary Figure 9. **Performance of a 105 mode Hermite-Gaussian sorter.** (a) Crosstalk and Efficiency of a 105 Hermite-Gaussian mode sorter (14 mode groups) using eight 340 × 340 pixels masks, and 8100 plane-waves per TM measurement. Shaded regions indicate the full range of results from these simulations. (b) Optimised masks after 500 mask updates.

In Supplementary Fig. 9 we show the simulated performance of our self-configuring algorithm for designing a 105 Hermite-Gaussian (HG) mode sorter. In this case we used 8 phase masks, each consisting of 340 × 340 pixels – chosen allowing just enough room to span two rows on the PLM used in our experiments. At this scale, it becomes more likely higher order modes to underperform relative to the average. To address this, we made a simple modification to our algorithm to include a heuristic weighting strategy to boost the contribution of higher order modes and bring their performance closer to the average. When calculating the mask update in Eq. 4 of the main paper, in this case we multiply each $\mathbf{s}_m^n$ by mode-dependent weights $w_n = \sqrt{n+b}$, where w_n were normalised according to $\sum_n (w_n)^2 = 1$. Here the parameter b controls the nonlinearity of the weighting: for large values of b, the weights w_n become nearly uniform, and for small values of b, higher order modes are boosted in the sum. In the MPLC from Supplementary Fig.. 9 we use $b = 15$.

It is worth noting that while the converged performance is comparable to the MPLCs shown in the main article, achieving reasonable levels of crosstalk and efficiency requires considerably more iterations due to the increased complexity of this MPLC design. Here, approximately 80 mask updates are needed to reach reasonable levels of performance.

In our current implementation, we estimate that roughly half of the total optimization time is spent changing the holograms (limited by the PLM refresh rate of 720 Hz or 1440 Hz), while the other half is consumed by software routines (e.g. digital holography, intra- and inter- TM drift correction). Here, for 80 mask updates, a total of 8480 TMs would need to be measured, each consisting of a $P = 90 \times 90 = 8100$ plane-wave basis plus approximately $r_{\text{drift}} = 8\%$ interleaved additional measurements for drift tracking. Excluding the time spent in software processing (i.e., setting $d_{\text{TM}} = d_{\text{mask}} = 0$), it follows from Eq. 5 in the main paper that each TM measurement would take approximately $t_{\text{TM}} \sim 6$ s at a hologram rate of $f = 1.44$ kHz, while the total acquisition time (main paper Eq. 6) $t_{\text{opt}} = t_{\text{TM}}(N+1) \cdot 80 \sim 14.3$ hours. Looking ahead, in the future development of this technology, a hologram rate of $f = 10$ kHz could reduce this time to just over 2 hours.

In such large-scale MPLC configurations, the computational burden becomes a significant bottleneck. However, as discussed in the main article, there are many low hanging fruits in optimising those routines, as many steps could be parallelized, and with this high mode count, these optimisations become increasingly worthwhile. On the same note, smarter sampling strategies for the TM, such as compressive sensing or sparsity-driven approaches, would also become more advantageous at this scale.

§10: Robustness of phase-drift correction protocol

In Supplementary Fig. 10(a), we plot example phase drift curves for the MPLC system shown in Fig. 1(c) within a single mask update. In this case the MPLC was optimised to simultaneously reshape $N = 3$ orthogonal speckle patterns into HG modes. The horizontal axis is indexed by the TM basis set, comprising 4096 plane-waves, where 340 phase drift measurements were interleaved uniformly throughout the acquisition. The curves in Supplementary Fig. 10(a,c) are obtained via nearest-neighbour interpolation of those 340 drift samples, and they reveal how the phase between the reference and signal arms varied during the TM measurement.

Supplementary Figure 10(b,d) show the corresponding phase difference curves between the columns of $\mathbf{T}_m'^n$ for $n = 1 \ldots N$, relative to columns of $\mathbf{T}_m'^{N+1}$, after applying the inter-TM drift correction. The transparent points here represent the phase of the summand in main paper Eq. 10 after inter-TM drift correction, while the solid lines represent the final result of Eq. 10. Notably, the curves in Supplementary Fig. 10 (a,c) were recorded hours apart, demonstrating the robustness of the drift correction protocol under different lab conditions. We also evaluated the system's performance in both cases, as shown in Supplementary

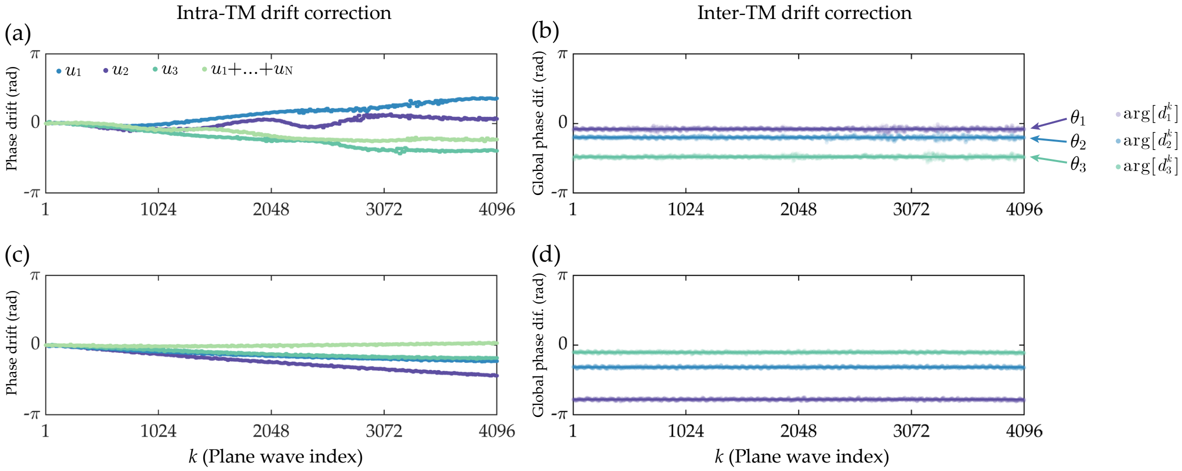

Supplementary Figure 10. **Representative intra-TM and inter-TM drift curves under different lab conditions.** (a,c) Phase drift variation along different Transmission Matrix (TM) measurements for a single mask update. Curves in (a,c) are used for intra-TM drift correction. (b,d) Residual phase difference (transparent points) and its corresponding average (solid curve) after inter-TM drift correction. (a, c) show the phase drift variation across a TM measurement. (b, d) show the residual phase difference between columns of $\mathbf{T}'^{n}_{m}$ (for $n = 1 \ldots N$) and the reference $\mathbf{T}'^{N+1}_{m}$ after inter-TM drift correction. Data in top and bottom rows were taken under different lab conditions several hours apart.

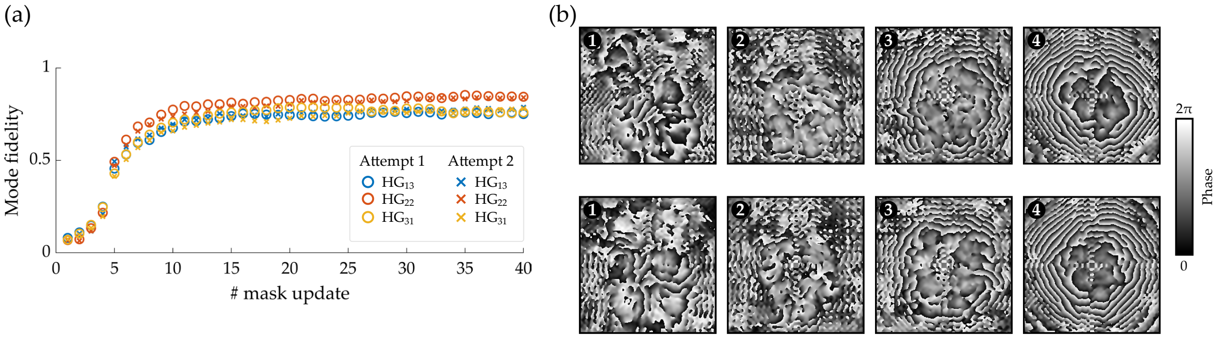

Supplementary Figure 11. **MPLC performance under different lab conditions.** (a) Mode fidelity for both MPLCs measured hours appart. (b) (top row) Optimised phase masks for the MPLC attempt 1, and (bottom row) attempt 2.

Fig. 11(a), with both attempts exhibiting similar results. In Supplementary Fig. 11(b), we display the optimized phase masks after 40 mask updates: the top row shows the masks from the first attempt, and the bottom row shows those from the second attempt. Furthermore, we observed that when the optimization is repeated immediately under near-identical conditions, it consistently converges to very similar phase masks with comparable performance, highlighting the robustness and repeatability of the optimization process itself.

We note that our phase drift correction protocol has no maximum phase drift limit – it can continue seamlessly throughout any range of global phase drift occurring between the reference arm and the beam transmitted through the MPLC. That said, there is a limit to the *rate* of phase drift we can accurately account for. In our current implementation we measure the relative drift at a rate of 60 Hz. This means we assume that the phase drift occurs on a timescale slower than 1/60 s. In our experiments (on a floated optical table) this condition was easily satisfied. If phase drift fluctuations may occur more rapidly, the drift measurement rate can be increased up to a maximum of half of the modulator rate (i.e., 720 Hz for our current PLM operating at 1.44 kHz.) at the expense of roughly doubling the total optimisation time (due to all of the extra drift correct measurements).

Looking to the future, for real-world operation, vibrations may also cause changes to laser pointing stability. If laser pointing direction changes appreciably within the measurement of a single TM measurement, then this may introduce substantial errors in the TM. However, when the self-configuring algorithm is running, then it will self-correct (in the following mask updates) for errors due to isolated vibrations causing an erroneous measurement of an individual TM. The main effect of these influences will be to slow convergence time. For example, we found that the optical table can be accidentally bumped and the algorithm

will automatically compensate for the resulting measurement errors. The combination of high-frequency global phase drift correction and self-configuration means that the system is highly resilient to external perturbations. Finally, to further improve the resilience to perturbations, we envision that instead of measuring the TM on each mask update, adaptive optimisation of each mask update would naturally self-correct for isolated external perturbations on a shorter timescale [9, 10].

§11: Self-configuring algorithm flowchart

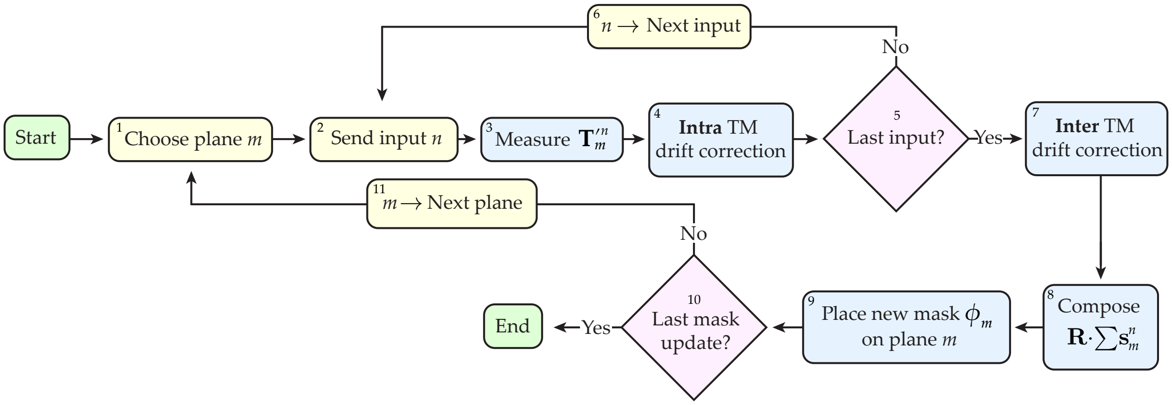

Supplementary Figure 12. **Self-configuring MPLC algorithm flowchart.** Flowchart illustrating the steps of the self-configuring multi-plane light conversion (MPLC) algorithm. The loops over input fields $n = 1 \dots N$ and planes $m = 1 \dots M$ can be performed in any order; in our experiments, both were executed sequentially in ascending order. In the input loop, we include an additional input $n = N + 1$, which corresponds to a superposition of the $n = 1 \dots N$ fields. The Transmission Matrix measurement of this superposition input is used as a reference for the inter-TM drift correction procedure.

Supplementary Figure 12 illustrates a high-level iterative flowchart of our self-configuring algorithm. Prior to starting the experiment, we only require a rough estimate of where the beam reflects from the PLM, and we assign each reflection an MPLC plane with index m. The algorithm begins by selecting one of these planes m (step 1) to optimise.

We then send a single input (step 2), shaped by the SLM in Fig. 3 and measure the Transmission Matrix (TM) of input n on plane m at the output (Cam 2) (steps 3 and 4). During each TM acquisition, we interleave drift measurements to enable subsequent intra correction of the TM. Example of phase-drift curves can be seen in in Supplementary Fig. 10(a,d). We iterate over all inputs ($n = 1$ to $N + 1$), where N is the number of orthogonal fields the MPLC is designed to support. The input $n = N + 1$ corresponds to a superposition of all inputs $n = 1$ to $n = N$, and is specifically used for the inter-TM drift correction procedure (step 7).

The order of inputs n is not strictly important, but in our experiments, we looped through them in ascending order. Once all TMs for inputs n have been measured, we use Eq. 10 from the main paper to drift-correct the $n = 1 \dots N$ TMs relative to the $n = N + 1$ reference.

At step 8, we compute the phase mask for the selected plane m using Eq. 4, and apply it to the plane. We then proceed to select another plane for optimization. Again, while the order of plane selection is flexible, in our experiments we optimized the planes sequentially in ascending order: $m = 1 \rightarrow N$, and repeated this until convergence was reached.

§12: Experimental factors limiting the performance of in-situ MPLC optimisation

Here we consider what limits the performance of our current experiments, and suggest potential future improvements to mitigate these factors:

(1) In our experiments, the number of TM samples (P) limits the highest spatial frequency of the MPLC masks. Increasing P (up to a maximum of the total number of pixels on each plane) would increase the resolution of the masks and thus improve the MPLC performance (see SI §2). However this also results in a longer optimisation time. Here strategies to reduce measurement overhead, as discussed in the 'Optimisation timescales' section of the main paper, will be explored in the future.

(2) The constrained field-of-view of our high-speed camera may also place a limit on the MPLC performance. For example, in order to run the camera at a rate that could keep up with the PLM switching rate, we had to reduce the field-of-view, meaning that after digital holographic processing, the resolution of the final fields was relatively low ($\sim 20 \times 20$ pixels). We think that using a camera that can accommodate a larger field-of-view at these high frame rates will improve the measurement fidelity of the output fields, and thus improve the design process.

(3) TM measurement noise will also contribute to inaccuracies in the mask update function. This effect could be reduced by

increasing the intensity of the laser used (there is ample scope for this as we used a low power 1 mW HeNe laser in our current experiments), and by using a more sensitive camera with a lower noise floor.
(4) Non-linear effects cannot be captured by a linear TM and so cannot be properly accounted for in our in-situ design process. The main source of non-linear effects is likely to be multiple reflections between the MEMS mirrors and the coverglass in front of them. Such multiple reflections result in a non-linear relationship between PLM mirror heights and the phase retardation of the reflected field. We emphasise that this nonlinearity is structural in nature [11, 12], preserving the wavelength, and should not be confused with optical non-linearities (e.g., harmonic generation). This is only a problem on the plane from which the TM is being measured – we do not have to be concerned with such multiple reflections on the other planes which are held static throughout TM measurement and so the effect of multiple reflections can still be captured by a linear matrix operator. We believe this effect is small, but would be further reduced by anti-reflection coating on the PLM coverglass. Furthermore, replacing TM measurement with adaptive optimisation [9, 10] would circumvent the need to assume a linear matrix and thus avoid the effect of such non-linearities.

[1] G. Orr, T. Byrum, M. Worrall, Z. Walker, W. McDonald, K. Oberascher, D. Doane, N. Gilly, S. O'Brien, P. Oden, *et al.*, High-volume production test methodology and parametrics of the texas instruments phase light modulator (plm), in *Emerging Digital Micromirror Device Based Systems and Applications XVII*, Vol. 13383 (SPIE, 2025) pp. 9–17.

[2] R. S. Ketchum and P.-A. Blanche, Diffraction efficiency characteristics for mems-based phase-only spatial light modulator with nonlinear phase distribution, in *Photonics*, Vol. 8 (MDPI, 2021) p. 62.

[3] J. C. Rocha, T. Wright, U. G. Būtaitė, J. Carpenter, G. S. Gordon, and D. B. Phillips, Fast and light-efficient wavefront shaping with a mems phase-only light modulator, Optics Express **32**, 43300 (2024).

[4] O. Lib, R. Shekel, and Y. Bromberg, Building and aligning a 10-plane light converter, Journal of Physics: Photonics **7**, 033001 (2025).

[5] V. López Pastor, J. Lundeen, and F. Marquardt, Arbitrary optical wave evolution with fourier transforms and phase masks, Optics Express **29**, 38441 (2021).

[6] W. Zhao, Z. Du, A. Zhai, and D. Wang, Wavefront imaging of a biological sample using dmd-based single-pixel phase-shifting interferometric techniques: An experimental comparison, Optics & Laser Technology **172**, 110483 (2024).

[7] B. Mastiani, G. Osnabrugge, and I. M. Vellekoop, Wavefront shaping for forward scattering, Optics express **30**, 37436 (2022).

[8] B. Mastiani, D. W. Cox, and I. M. Vellekoop, Practical considerations for high-fidelity wavefront shaping experiments, Journal of Physics: Photonics **6**, 033003 (2024).

[9] I. M. Vellekoop and A. Mosk, Phase control algorithms for focusing light through turbid media, Optics communications **281**, 3071 (2008).

[10] D. B. Conkey, A. N. Brown, A. M. Caravaca-Aguirre, and R. Piestun, Genetic algorithm optimization for focusing through turbid media in noisy environments, Optics express **20**, 4840 (2012).

[11] F. Xia, K. Kim, Y. Eliezer, S. Han, L. Shaughnessy, S. Gigan, and H. Cao, Nonlinear optical encoding enabled by recurrent linear scattering, Nature Photonics , 1 (2024).

[12] M. Yildirim, N. U. Dinc, I. Oguz, D. Psaltis, and C. Moser, Nonlinear processing with linear optics, Nature Photonics , 1 (2024).
