# Supplementary material for: Self-configuring high-speed multi-plane light conversion
Source: Nat Commun. 2025 Dec 8;17:73. doi: 10.1038/s41467-025-66798-2 (PMC12770535; doi:10.1038/s41467-025-66798-2)
Supplement: Supplementary file 2 — Description of Additional Supplementary Files [file 41467_2025_66798_MOESM2_ESM.pdf]

Description of Additional Supplementary Files:

Supplementary movie 1: The experimentally measured output field as a function of mask update number throughout the MPLC in-situ optimisation process. Here the input field is a speckle pattern, and the target output field is a Laguerre-Gaussian beam LG11 of vortex charge $\ell = +1$ and radial index $p = 1$. See also main paper Fig. 1(b).

Supplementary movie 2: The 3 different experimentally measured output fields generated when the MPLC is illuminated with the 3 different input fields, as a function of mask update number throughout the MPLC in-situ optimisation process. Here the input fields are 3 orthogonal speckle patterns, and the output fields are Hermite-Gaussian beams of mode order HG13, HG22 and HG31. See also main paper Fig. 1(c).
